# Pretreatment inflammatory indices predict Bevacizumab response in recurrent Glioma

**DOI:** 10.20517/cdr.2020.33

**Published:** 2020-08-07

**Authors:** Alicia Martínez-González, Raquel Cabrera, Marta Lloret, Pedro C. Lara

**Affiliations:** ^1^Department de Matemáticas, Universidad de Castilla-La Mancha, ETSI Industriales, Avda, Ciudad Real 13071, Spain.; ^2^Oncología Radioterápica Hospital Universitario de Gran Canaria Dr Negrin, Barranco de la Ballena s/n, Las Palmas de Gran Canaria, Las Palmas 35010, Spain.; ^3^Facultad de Ciencias de la Salud, Universidad de Las Palmas de Gran Canaria, Paseo de Blas Cabrera Felipe, s/n, Las Palmas de Gran Canaria, Las Palmas 35016, Spain.; ^4^Fundación Canaria del Instituto Canario de Investigación del Cáncer, Avda de la Trinidad 61 Torre Agustín Arevalo 7 planta La Laguna, Santa Cruz de Tenerife 38204, Spain.; ^5^San Roque University Hospitals, Dolores de la Rocha, 5, Las Palmas de Gran Canaria, Las Palmas 35001, Spain.; ^6^Fernando Pessoa Canarias University, Dolores dela Rocha 14, Las Palmas de Gran Canaria, Las Palmas 35016, Spain.

**Keywords:** Recurrent glioma, bevacizumab, predictive biomarker, inflammatory indices, lymphocytes

## Abstract

**Aim**: It remains unclear what the best therapeutic option for recurrent glioma patients after Stupp treatment is. Bevacizumab (BVZ) is commonly administered in progression, but it appears that only some patients benefit. It would be useful to find biomarkers that determine beforehand who these patients are.

**Methods**: The protocol included 31 high-risk progressing glioma patients after Stupp treatment who received BVZ 5-10 mg/kg every 14 days and temozolomide (3-19 cycles, 150-200 mg five days each 28-day cycle) during a mean of eight cycles of BVZ or until tumor progression or unacceptable toxicity. We analyzed the clinical outcome values of inflammatory indices measured before BVZ administration.

**Results**: Lymphocyte level before BVZ administration was the best independent predictor of overall survival (HR = 0.34; 95%CI: 0.145-0.81; *P* = 0.015). The area under the receiver operating characteristic (ROC) curve was 0.823, with 1.645 being the optimal cut-off value, and 0.80 and 0.85 the sensitivity and specificity values, respectively. Responder and non-responder survival curves were also significantly different, considering the first and second tertiles as cut-off points. The number of BVZ cycles was not related to lymphopenia. Pretreatment neutrophil, platelet levels, platelet-to-lymphocyte ratio (PLR), and neutrophil-to-lymphocyte ratio (NLR) did not have independent predictive value. Inflammatory variables were not correlated with each other. However, patients with high NLR and PLR simultaneously (double positive PLR-NLR) showed a worse clinical outcome than the rest (*P* = 0.043).

**Conclusion**: Pretreatment lymphocyte levels and double positive PLR-NLR could be used as non-invasive hematological prognostic markers for recurrent gliomas treated with bevacizumab. A close relationship emerged between inflammation and angiogenesis.

## Introduction

Primary brain tumors constitute about 2% of all malignant tumors; half of them are high-grade gliomas^[1,2]^ and some low-grade gliomas show aggressive transformation, becoming high-grade gliomas, including glioblastoma (GBM)^[3]^. GBM aggressiveness leads to a median survival time of around three months without treatment, and 12-15 months with all therapeutic interventions^[4]^. Only 8%-12% of patients survive for two years and fewer than 3% of patients survive for five years^[1,5-7]^. Standard treatment includes maximal safe surgical resection and the Stupp protocol: concomitant temozolomide (TMZ) (75 mg seven days/week) to radiotherapy (RT) and adjuvant TMZ (six cycles, 150-200 mg five days each 28-day cycle). Progression and recurrence are observed with a median time of 10 weeks after finishing the standard treatment^[1]^. Tumor progression is associated with deterioration of neurocognitive function and a decrease in quality of life^[5]^. When progression occurs after the Stupp protocol, there is no standard treatment^[8]^. Therapeutic options then depend on tumor and patient characteristics: second surgery, re-irradiation, or chemotherapy such as TMZ, bevacizumab (BVZ), fotemustine, lomustine, cyclophosphamide, or irinotecan^[9]^. GBM is characterized by increased expression of vascular endothelial growth factor (VEGF), which stimulates angiogenesis^[8,10-12]^. BVZ is a humanized monoclonal immunoglobulin G1 which binds to VEGF. BVZ may reduce neo-vasculature, improve blood vessel integrity, and increase the percentage of apoptotic cells^[9,13]^. Since BVZ can only cross the blood–brain barrier in disrupted areas^[14]^, it is associated with non-enhancing tumor progression, improvement of clinical performance, and progression-free survival^[15,16]^. BVZ remains one of the few Food and Drug Administration (FDA) approved and most commonly prescribed therapies for recurrent GBM. However, the reduction in tumor size following therapy is transient and it is unclear which patients are most likely to benefit from treatment^[17,18]^.

GBM patient prognosis mainly depends on treatment administered, age, and Methyl Guanine Methyl Transferase (MGMT*)* methylation. Other markers seem to be related to survival: sex, Karnofsky Performance Status (KPS), Mini-Mental score, tumor location, necrosis, contrast-enhancement on preoperative Magnetic Resonance Image (MRI), or MRI texture variables^[19-22]^. A histopathological, molecular, immunohistochemical, and neuroradiological study did not find any predictive marker of response or survival benefit for BVZ in GBM^[23]^.

As generalized indicators of inflammation, the complete blood cell counts and their ratios have been investigated as biomarkers in several cancers and they have been found to be very accessible prognostic markers^[24-29]^. High lymphocytic infiltration is associated with improving survival and superior response to systemic therapy, whereas a low peripheral blood lymphocyte count is related to poor cancer prognoses^[26]^. Neutrophil infiltration stimulates tumor growth, angiogenesis, and metastasis through the secretion of VEGF, while platelets can also support cancer progression by promoting proliferation, angiogenesis and metastasis^[24-26]^. Preoperative neutrophil-to-lymphocyte ratio (NLR) could reflect tumor burden and clinical outcomes in ovarian cancer, pancreatic cancer, renal cell carcinoma, and colorectal carcinoma. The prognostic value of NLR was the highest in mesothelioma and pancreatic cancer. Prognostic value of platelets-to-lymphocyte ratio (PLR) has been evaluated in colorectal, gastroesophageal, hepatocellular, pancreatic, and ovarian cancer^[27-29]^. NLR was significantly associated with improved results after addition of BVZ in a previous metastatic colorectal study.

However, we know less about the roles that inflammatory indices play as biomarkers for GBM patients^[30-33]^. NLR showed prognostic impact for GBM to first progression in patients undergoing second surgery^[30-32]^. NLR and PLR are considered prognostic markers in new gliomas: the higher are the ratios, the worse is the tumor prognosis. Pretreatment NLR is of prognostic significance independent of MGMT status and is superior to PLR as a prognostic factor^[33]^. Some authors have suggested a decision-tree-based model for recurrent GBM but peripheral blood information and response to BVZ were not considered^[34]^.

There are few studies focused on prognostic inflammatory biomarkers for recurrent gliomas and even fewer focusing on BVZ response. Only patients with a high neutrophil count benefited from BVZ in an exploratory cohort of patients treated with radio-chemotherapy, and then BVZ or chemotherapy at recurrence. BVZ use increased median overall survival (OS) (18.7 months *vs.* 11.3 months, *P* = 0.0014)^[35]^. However, a recent study found that neutrophils did not have predictive value for recurrent gliomas treated with BVZ. This study suggested the use of blood count changes over the entire duration of BVZ therapy to predict response. Changes in lymphocyte counts predicted survival time from BVZ initiation to death^[35,36]^. Nevertheless, it would be desirable to find a predictive biomarker obtained before treatment begins. To our knowledge, no one has analyzed NLR and PLR with survival in recurrent gliomas treated with BVZ.

In this study, we analyzed serum levels before treatment with BVZ to determine whether lymphocytes, neutrophils, platelets, NLR, or PLR can be proposed as prognostic markers of overall survival in recurrent gliomas treated with BVZ. The final aim was to classify patients who receive more benefit from the treatment with BVZ, considering new immune markers.

## Methods

### Patients

Data on 31 patients with recurrent glioma were retrospectively collected and analyzed. Clinical variables such as age, sex, preoperative KPS, extent of resection, localization, histopathology, and adjuvant therapy were included. After first surgery, most patients received radiotherapy (93.55%) and concomitant temozolomide (80.64%). Patients had follow-ups with serial MRI, so progression was defined as clinical progression, radiological progression, or both. Tumor progression was defined according to the modified World Health Organization (WHO) criteria as an appearance of new lesions, an increase in tumor size by 25% in radiological images, or an increased need for corticosteroids^[32]^. The median time from diagnosis of high-grade glioma to recurrence was 15 months. From progression diagnosis, six patients underwent surgery, five of whom were GBM at baseline and the other had a grade II astrocytoma that progressed to GBM. Patients who did not receive surgery were categorized as their previous histological subtype and only two patients were irradiated. This study was carried out in accordance with the protocol “PATRORA” approved by the Agencia Española de Medicamentos y Productos Sanitarios (Ref. PLJ-BEV-2016-01) and by the Hospital Universitario de Gran Canaria Doctor Negrín committee (Ref. 170007).

### Inflammatory data and toxicity

The levels of neutrophils, platelets, and lymphocytes were measured. To study the association between inflammatory response and recurrent gliomas, median and tertile levels of neutrophils, lymphocytes, and platelets were used as cut-off values to discriminate between groups.

NLR and PLR were calculated by dividing the absolute neutrophil or platelet counts, respectively, by the absolute lymphocyte count, using the hemogram prior to bevacizumab treatment. Median NLR and PLR values were used to classify patients into positive (above the median) or negative (below the median) NLR and PLR groups, respectively. Patients with positive NLR and PLR simultaneously were included in the double-positive group while those with negative NLR and PLR simultaneously were in the double-negative group. In any other case, the patient was considered as single positive.

### Statistical analysis

Statistical analysis was performed using Statistical Package for The Social Science software (SPSS). For all analyses, a *P*-value < 0.05 was accepted as significant. First, the Kolmogorov-Smirnov test was used to identify whether the variables followed a normal distribution to choose a parametric or non-parametric test. Comparisons among groups were performed using the *c*^2^ test for categorical variables, and Kruskal-Wallis and Mann-Whitney tests for continuous variables. *c*^2^ and T-student tests were used to compare the distribution differences of each variable. Correlation between variables was performed using the Spearman test. Survival analysis was evaluated through Kaplan-Meier curves and comparison between subgroups was reproduced by a Log-Rank probability test. Cox proportional hazards regression analysis was used to obtain the HR and its adjusted 95%CI for each threshold. The area under the receiver operating characteristic (ROC) curve was calculated to evaluate optimal cut-off values.

## Results

### Descriptive data analysis

After Stupp treatment, all patients received BVZ 5-10 mg/kg every 14 days and 94% combined the BVZ with temozolomide (3-19 cycles, 150-200 mg five days each 28-day cycle) during a mean of eight cycles of BVZ or until tumor progression or unacceptable toxicity. Fifty-five percent received temozolomide again in accordance with the Stupp protocol, five days every 28 days at a dose of 150-200 mg/m^2^ and 38% a metronomic daily dose of 40-60 mg. Table 1 summarizes the main patient features included in the study. In fact, 55.17% of the patients received Stupp treatment after progression (4.5 average cycles); however, this did not affect later neutropenia.

**Table 1:** Patients features in diagnosis and progression

		1st diagnosis		Progression	
		No.	%	No.	%
Age (median in years)		46		48	
Progression diagnosis	Radiological			11	32.26
	Clinical			1	
	Clinical-radiological			19	67.74
KPS	Median	90		85	
	KPS > 80	27	87.09	14	45.16
	KPS ≤ 80	4	12.90	14	45.16
Location	Frontal	4	12.90	4	12.90
	Temporal	4	12.90	3	9.68
	Parietal	3	9.68	5	16.13
	Occipital	4	12.90	5	16.13
	Diencephalon	2	6.45	1	3.22
	Cerebellum	1	3.22	1	3.22
	Overlapping	13	41.94	12	38.7
Edema		27	87.09	20	67.74
Surgery	Total	30	96.77	6	19.35
	Total resection	15	48.39	2	33.33
	Subtotal resection	13	41.94	4	66.67
	Biopsy	2	6.45	0	0
Gliadel		10	32.26	5	83.33
Anatomic pathology	Astrocytoma II	3	9.67	2	6.66
	Oligodendroglioma II	1	3.23	1	3.33
	Glioma II	1	3.23	1	3.33
	Oligodendroglioma III	2	6.45	2	6.66
	Astrocytoma III	5	16.13	5	16.66
	GBM and High-grade glioma	18	58.06	19	63.33
RT		29	93.55	2	6.45
Concomitant TMZ		25	80.64	2	6.45
Adjuvant TMZ	Total	23	74.19	29	93.55
	Stupp	23	74.19	16	55.17
	Cycles (mean)	7.43		4.38	
	Confidence interval (95%)	(5.64–9.23)		(2.34–6.41)	
	Metronomic (any time)	6	26	13	44.83

KPS: karnofsky performance status; No.: number of patients; RT: radiotherapy; TMZ: temozolomide

**Figure 1:**
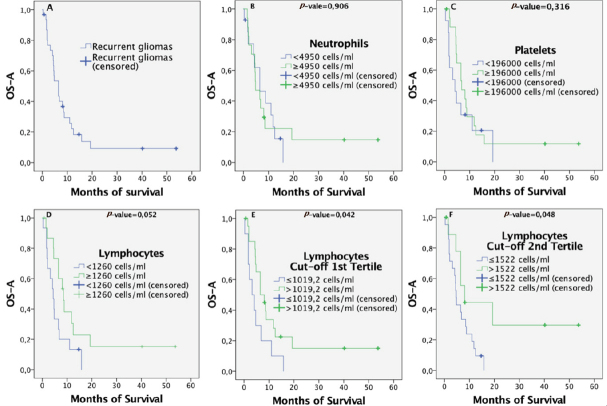
Overall survival from BVZ administration calculated by Kaplan-Meier curves for the total of 31 patients: (A) all patients in the same group; (B) stratified by levels of neutrophils; (C) stratified by levels of platelets; (D-F) stratified by levels of lymphocytes using the median = 1260 cells/mL, the first tertile = 1019 cells/mL, and the second tertile = 1522 cells/mL as the cut-offs, respectively. BVZ: bevacizumab

At the end of the study, 26 patients had died (83.8%), 2 continued receiving bevacizumab cycles as they were in a state of remission, and 1 patient showed a response above 50%. The median survival from BVZ administration was 6.5 months (95%CI: 4.1-8.9 months) and long-term survivals represented 9.6% (living more than three years). Figure 1A displays the cumulative survival curve from BVZ administration for all 31 recurrent gliomas.

Hemogram pre- and post-bevacizumab treatment determined toxicity: levels of anemia (less than 8 g/dL) appeared in nine patients (29%), lymphopenia was experienced by 54.8%, and 19% showed severe lymphopenia (less than 500 lymphocytes/mL). Twenty-nine percent had thrombocytopenia with lower than 50,000 platelets/mL. Neutropenia appeared in only one patient. Table 2 shows the mean, median, standard deviation, and 95%CI for levels of neutrophils, lymphocytes, platelets, NLR, and PLR.

**Table 2:** Levels of neutrophils, lymphocytes, platelets (mg/dL), NLR, and PLR blood counted before first cycle of bevacizumab

	Neutrophils (mg/dL)	Lymphocytes (mg/dL)	Platelets (mg/dL)	NLR	PLR
Mean	5.67	1.26	222.03	5.71	207.19
Median	4.95	1.26	196.00	3.97	186.42
SD	2.92	0.50	85.35	6.00	132.98
95%CI	(4.6-6.75)	(1.08-1.45)	(190.73-253.34)	(3.51-7.91)	(158.40-255.97)

SD: standard deviation; NLR: neutrophil-to-lymphocyte ratio; PLR: platelet-to-lymphocyte ratio

### Pre-bevacizumab levels of neutrophils or platelets were not related to survival

The concentrations were considered low if they were below the median, 4950 cells/mL for neutrophils and 196,000 cells/mL for platelets. The concentrations were considered high in other cases. We did not find significant differences in overall survival between patients with high and low concentration of neutrophils or platelets. Figure 1B and C shows their survival curves after BVZ treatment with *P*-values of 0.906 and 0.316 for neutrophils and platelets, respectively.

### Patients with high Pre-bevacizumab levels of lymphocytes showed better outcomes

Patients with levels of lymphocytes above the median (1260 cells/mL) tended to show better outcomes. This association was close to significance with *P*-value = 0.052, as shown in Figure 1D. However, both the first (1019 cells/mL) and second tertiles (1522 cells/mL) as cut-off thresholds for lymphocytes showed significant survival differences (with *P*-value = 0.042 and *P*-value = 0.048, respectively). Low levels of lymphocytes were associated with worse outcomes, with *P* = 0.042 in Figure 1E and *P* = 0.048 in Figure 1F, choosing the first and second tertiles, respectively.

### Pre-bevacizumab NLR or PLR ratios were not individually related to survival

**Figure 2:**
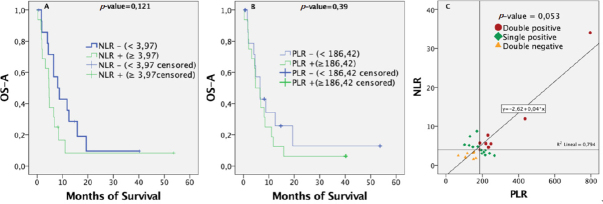
Overall survival from BVZ administration calculated by Kaplan-Meier curves for 31 patients stratified by: (A) NLR positive or negative; (B) PLR positive or negative; (C) Association between NLR and PLR. It seems the more NLR increases, the more PLR increases as well. This association is almost statistically significant following a Spearman test (*P* > 0.05; 95%CI). BVZ: bevacizumab; NLR: neutrophil-to-lymphocyte ratio; PLR: platelet-to-lymphocyte ratio

Patients were classified as negative for NLR if their value was below the median (3.97). Similarly, patients were considered negative for PLR if their value was below the median (186.42). Both negative groups (for NLR or PLR) tended to show better prognoses, with survival curves always above their respective positive groups. Nevertheless, Figure 2A and B shows how these differences between positive and negative groups were not significant (*P*-values of 0.121 for NLR and 0.39 for PLR). The same association was made using tertiles as cut-off thresholds, with similar results. Pre-bevacizumab NLR and PLR ratios were not individually related to survival.

### Correlation between inflammation variables

Lymphocytes were correlated with platelets (*P* = 0.042), NLR (*P* = 0.025), and PLR (*P* < 0.001) only weakly (correlation coefficient 0.368, -0.401, and -0.589, respectively). As expected, neutrophils were correlated with NLR (*P* > 0.001 and correlation coefficient of 0.676) and platelets with PLR (*P* < 0.020 and correlation coefficient of 0.415). Figure 2C shows that the more NLR increases, the more PLR increases; this association was not statistically significant according to the Spearman test (*P*-value = 0.053; 95%CI).

### Combined information PLR-NLR had predictive value

**Figure 3:**
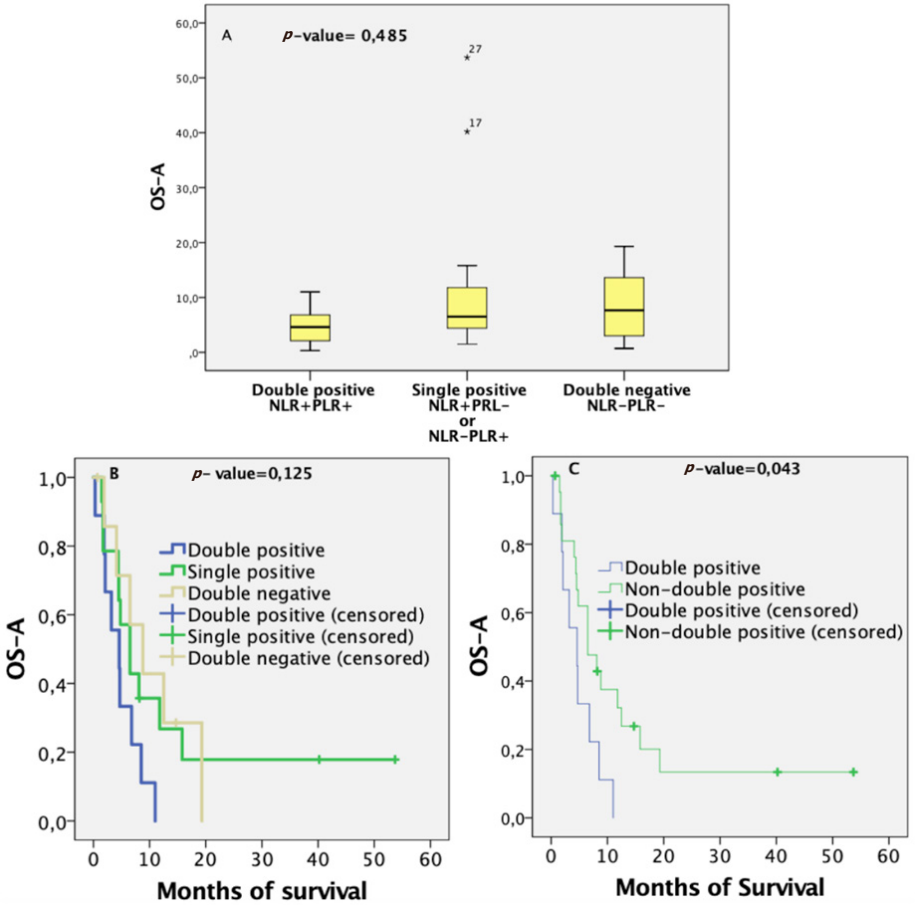
Predictive survival ability combining PLR-NLR information: (A) diagram boxes comparing median survival after BVZ administration among the three groups; (B) overall survival from BVZ administration calculated by Kaplan-Meier curves for 31 patients stratified into three groups, namely double positive, double negative, and single positive; (C) overall survival from BVZ administration calculated by Kaplan-Meier curves for 31 patients stratified into a double-positive group and otherwise. BVZ: bevacizumab; NLR: neutrophil-to-lymphocyte ratio; PLR: platelet-to-lymphocyte ratio

As association between PLR and NLR was almost significant, we suggest a new patient classification based on both values simultaneously. According to these two variables, patients belong to one of three groups: double positive (both values above their median), double negative (both values below their median), or single positive (the other cases). As the survival of patients with one ratio over the median and the other below it was similar, we classified them into the same group. Median survival was highest for the double-negative group and lowest for the double-positive group. The survival distribution and its median are displayed in Figure 3A. However, statistical difference between the three groups in terms of mean survival was not found using the Kruskal-Wallis test (*P*-value = 0.485; 95%CI). Figure 3B displays the survival curves after BVZ treatment for the three groups with *P*-value = 0.125. However, comparing the double-negative and single-positive groups, a significant survival difference was observed. The double-positive group had significantly worse prognosis (*P*-value = 0.043, 95%CI) than the non-double-positive group, as shown in Figure 3C.

### Similar toxicity for patients classified by combining PLR-NLR information

The median age was 40.5, 52, and 48 years for the double-negative, single-positive, and double-positive groups, respectively. Although other therapies had been provided previously, Table 3 shows that toxicity was similar in the three groups. RT or TMZ did not influence NLR and PLR values. We focused on neutropenia and thrombocytopenia before BVZ administration and the differences were not significant: 25% of the double-negative group, 11.1% of the double-positive group, and 7% of the single-positive group had neutropenia *P*-value of 0.849 stratifying into double positive *vs*. non-double positive). Moreover, 62% of the double-negative group had thrombocytopenia, whereas 22% and 28.6% appeared in the double-positive and single-positive groups, respectively (*P*-value of 0.324 stratifying into two groups). Neither NLR nor PLR was related to hematologic BVZ toxicity: 22% and 21% of double-positive group and single-positive group had neutropenia and not a single case of double-negative had it (*P*-value of 0.555 stratifying into two groups). Fifty percent of double-negative group, 44% of double-positive group, and 28.6% of single-positive group developed thrombocytopenia (*P*-value 0.675 stratifying into two groups).

**Table 3:** Clinical characteristics of patients according to PLR-NLR groups

	Double Positive (*n* = 9)	Single Positive (*n* = 14)	Double Negative (*n* = 8)	*P*-value (3 groups)	*P*-value (2 groups)
Pre-BVZ toxicity	(Yes/No)	(Yes/No)	(Yes/No)	0.984	0.873
Anemia	3/6	5/9	3/5		
Neutropenia	1/8 (11.1%/88.9%)	1/13 (7%/93%)	2/6 (25%/75%)	0.477	0.849
Lymphopenia	5/4	11/3	7/1	0.285	0.129
Leukopenia	1/8	2/12	2/6	0.716	0.627
Thrombocytopenia	2/7 (22%/78%)	4/10 (28.6%/71.4%)	5/3 (62.5%/37.5)	0.171	0.324
Post-BVZ toxicity	(Yes/No)	(Yes/No)	(Yes/No)	0.203	0.675
Anemia	4/5	7/7	1/7		
Neutropenia	2/7 (22%/78%)	3/11 (21%/78.5%)	0/8 (0%/100%)	0.354	0.555
Lymphopenia	7/2	10/4	5/3	0.786	0.593
Leukopenia	1/8	1/13	0/8	0.642	0.499
Thrombocytopenia	4/5 (44%/56%)	4/10 (28.6%/71.4%)	4/4 (50%/50%)	0.560	0.675

NLR: neutrophil-to-lymphocyte ratio; PLR: platelet-to-lymphocyte ratio; BVZ: bevacizumab; 3 groups: double positive, single positive, or double negative; 2 groups: double positive *vs.* non-double positive

### Lymphocyte level was the best independent predictor

Lymphocyte level before BVZ administration was the best independent predictor of overall survival, as displayed in Table 4 (HR = 0.34; 95%CI: 0.145-0.81; *P* = 0.015). The area under the ROC curve was 0.823 and the optimal cut-off value was 1645 cell/mL (0.80 sensitivity and 0.85 specificity). Interestingly, the number of BVZ cycles was not related to lymphopenia, and differences in the distribution of age, sex, KPS, extent of resection, alcohol/tobacco use, and histological diagnoses were not significant.

**Table 4:** Uni- and multivariable analysis (Cox regression) for the most representative variables associated with OS after BVZ treatment

Variables	OS-A (univariable)		OS-A (multivariable)	
	HR, (95%CI)	*P*-value	HR, (95%CI)	*P*-value
Age as continuous variable	1.02 (0.96-1.05)	0.306		
Gender	1.25 (0.52-2.98)	0.619		
Neutrophil Level (cells/mL)	1.03 (0.89-1.19)	0.704		
Median: < 4950 *vs*. ≥ 4950	0.95 (0.43-2.11)	0.906		
Platelet Level (cells/mL)	1.00 (0.99-1.00)	0.145		
Median: < 196,000 *vs*. ≥ 196,000	0.33 (0.68-3.23)	0.316		
Lymphocyte Level (cells/mL)	0.34 (0.145-0.81)	0.015*		
Median: < 1260 *vs*. ≥ 1260	2.15 (0.97-4.80)	0.052		
First tertile: < 1019 *vs*. ≥ 1019	2.25 (1.00-5.04)	0.042*		
Second tertile: < 1522 *vs*. ≥ 1522	2.62 (0.96-7.10)	0.048*		
NLR	1.04 (0.99-1.10)	0.105		
Median: (< 3.97 *vs*. ≥ 3.97)	0.54 (0.24-1.20)	0.121		
PLR	1.00 (1.00-1.00)	0.249		
Median: (< 186.42 *vs*. ≥ 186.42)	0.71 (0.32-1.56)	0.390		
PLR-NLR (Double positive *vs*. non-double positive)	2.35 (0.99-5.56)	0.043*		
Neutropenia	0.93 (0.32-2.72)	0.890		
Lymphopenia	2.23 (0.82-6.10)	0.118		
Leukopenia	0.77 (20.29-2.06)	0.604		
Thrombocytopenia	1.20 (0.52-2.73)	0.682		
Anemia	2.22 (0.92-5.33)	0.076		
Lymphocyte Level (cells/mL)			0.42 (0.16-1.10)	0.078*
PLR-NLR (Double positive *vs*. non-double positive)			1.50 (0.55-4.04)	0.436

*P-values significant. OS: overall survival; BVZ: bevacizumab; OS-A: overall survival from bevacizumab administration; HR: hazard ratio; NLR: neutrophil-to-lymphocyte ratio; PLR: platelet-to-lymphocyte ratio

### Lymphocyte level and outcome depending on tumor localization

**Figure 4:**
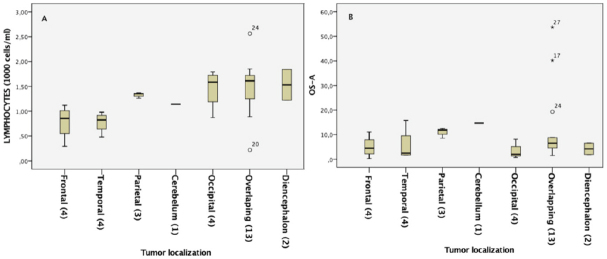
Lymphocyte levels and outcomes with patients classified by tumor localization: (A) lymphocyte level diagram box depending on tumor localization; (B) diagram box of overall survival from BVZ administration depending on tumor localization. BVZ: bevacizumab

Figure 4 shows seven different localizations assessed using MRI: frontal (four patients), temporal (four patients), parietal (three patients), cerebellar (one patient), occipital (four patients), overlapping (thirteen patients), and diencephalon (two patients). Figure 4A exhibits the distribution of pretreatment lymphocyte levels depending on tumor localization. Patients with frontal or temporal tumors tended to show lower lymphocyte values. Figure 4B shows the OS after BVZ treatment for the same groups of patients. The three patients with the best outcome were found in the overlapping group. The parietal group had a high lymphocyte level but also a high OS with a small standard deviation in both cases. No statistical differences were found between patients with tumors in the left and right hemispheres, in terms of either lymphocyte levels or survival.

### Limitations

The main limitations of our study are its retrospective design and the small and diverse sample.

## Discussion

Low-grade gliomas may undergo aggressive transformation, becoming high-grade gliomas, and they also tend to recur after a few months^[37]^. It is still unclear what the best therapeutic option for those recurrent glioma patients who have already received the standard radio- and chemotherapy actually is. Scientists are trying to find: (1) new treatments to prolong OS and quality of life; and (2) biomarkers that predict the response to these treatments. The FDA approved BVZ as a single agent for treatment of recurrent GBM with poor or refractory response to other therapies^[17]^. The European Medicines Agency rejected this indication, although some studies have questioned the decision^[18]^. No new agents have been approved for second-line therapy of GBM in Europe since the approval of TMZ in 1999. It has been reported that BVZ enhances the effect of RT and TMZ, allowing chemotherapy and oxygen to better perfuse within the tumor^[14]^. BVZ can attenuate tumor-associated brain edema and thus improve patient symptoms due to a reduction in steroid use^[10]^. Nevertheless, it seems that only some patients benefit; thus, it would be useful to find biomarkers that determine those who might respond beforehand^[18]^.

Inflammation and cancer are linked: high levels of neutrophils and platelets are related to poor prognosis for several cancers while high lymphocytic infiltration is associated with improved survival and superior response to systemic therapy^[25,28]^. However, there are few studies focused on prognostic inflammatory biomarkers for recurrent gliomas^[30-35]^ and even fewer focused on BVZ response after recurrence.

To our knowledge, no one has analyzed the pretreatment levels of inflammatory indices with survival in recurrent gliomas treated with BVZ. We analyzed the correlation between survival and inflammatory components measured before BVZ administration in 31 recurrent gliomas: neutrophils, lymphocytes, platelet counts, the ratios of NLR and PLR, and the combination PLR-NLR.

Our results suggest that pre-BVZ levels of neutrophils and platelets do not seem to act as biomarkers. However, the group of patients with high levels of lymphocytes lived longer than the others. This result is robust since several cut-off values showed significant differences between groups. We recommend using a threshold for lymphocytes between 1019 cells/mL (first tertile) and 1522 cells/mL (third tertile). Then, we do not know if lymphocyte infiltration before BVZ treatment could improve overall survival for recurrent glioma patients.

Since lymphocytes were the most powerful biomarker, we analyzed if it was related to the location of the tumor. Those patients with frontal or temporal tumors tended to have lower lymphocyte values. The three patients with the best outcome were found in the overlapping group. The parietal group had a high lymphocyte level but also a high OS with small standard deviation in both cases. Unfortunately, the low number of individuals per group did not allow us to determine differences between groups. However, these preliminary results could inspire other groups and motivate a deeper study.

The relation between survival and pretreatment NLR or PLR was not statistically significant and survival curves where similar (*P*-value = 0.121 and *P*-value = 0.39, respectively). The Spearman correlation p-value between NLR and PLR was 0.053. We would expect to have a more significant correlation value with a larger number of patients. It is noteworthy that the survival curve for NLR-PLR positive patients was below the survival curve of NLR-PLR negative patients. This result fits very well with previous results that a NLR > 4 prior to second surgery was a poor prognostic factor in GBM, and later progression was associated with longer survival in patients but not in longer survival after second surgery^[32]^.

The combined inflammatory information PLR-NLR may affect the evolution of high-grade gliomas at recurrence treated with bevacizumab. In fact, the double-positive category is associated with the worst prognosis (*P*-value = 0.043). This merger had not been carried out in the studies that were previously reviewed. We hypothesize that patients with double-positive PLR-NLR have more angiogenesis and the BVZ effect is smaller, having worse prognosis. Nevertheless, a deeper study would be necessary.

Regarding toxicity, we found fewer patients suffering lymphopenia and leukopenia with treatment at recurrence than at diagnosis. However, more patients had anemia, neutropenia, or thrombocytopenia at recurrence. Some of them also received other therapies after progression such as second surgery or TMZ. Although 55.17% of patients received Stupp treatment after progression (4.5 average cycles), later neutropenia was not affected. A complication when studying recurrence is the possible modification of our results due to previous administration of therapies or development of infections. In principle, the toxicity the patients experience in prior therapy could influence posterior NLR and PLR levels. It is believed that patients with more toxicity respond better to therapy. In our study, toxicity was similar in all groups: neither NLR nor PLR was related to hematological BVZ toxicity.

This study classified recurrent glioma patients who receive more benefit from the treatment with BVZ considering new immune markers: both lymphocyte levels and the combination of PLR-NLR could be employed as non-invasive hematological prognostic markers for recurrent high-grade gliomas treated with BVZ. However, lymphocyte level before BVZ administration was the best independent predictor of overall survival from bevacizumab administration (OS-A). A close relationship appeared between inflammation and angiogenesis, although this is not very well understood yet.

## Declarations

### Copyright

© The Author(s) 2020.
